# Avatars in the educational metaverse

**DOI:** 10.1186/s42492-025-00196-9

**Published:** 2025-06-10

**Authors:** Md Zabirul Islam, Ge Wang

**Affiliations:** 1 Department of Computer Science, School of Science, Rensselaer Polytechnic Institute, NY 12180 Troy, United States; 2 Department of Biomedical Engineering, School of Engineering, Rensselaer Polytechnic Institute, NY 12180 Troy, United States

**Keywords:** Avatars, Metaverse, Artificial intelligence, Virtual environments, Data security

## Abstract

Avatars in the educational metaverse are revolutionizing the learning process by providing interactive and effective learning experiences. These avatars enable students to engage in realistic scenarios, work in groups, and develop essential skills using adaptive and intelligent technologies. The purpose of this review is to evaluate the contribution of avatars to education. It investigated the use of avatars to enhance learning by offering individualized experiences and supporting collaborative group activities in virtual environments. It also analyzed the recent progress in artificial intelligence, especially natural language processing and generative models, which have significantly improved avatar capabilities. In addition, it reviewed their use in customized learning, contextual teaching, and virtual simulations to improve student participation and achievement. This study also highlighted issues impacting its implementation, including data security, ethical concerns, and limited infrastructure. The paper ends with implications and recommendations for future research in this field.

## Introduction

The rapid advancement of digital technology has transformed education, providing many opportunities for interactive and immersive learning. One such innovation is the metaverse, a shared three-dimensional (3D) virtual space, which has drawn attention for its potential to change education. The metaverse enhances learning experiences and reduces the need for physical travel. It facilitates the exchange of practices and ideas by overcoming geographical and temporal barriers and supporting eco-friendly educational solutions [1].

A key criterion for sustainable schools is the selection of locations that minimize exposure to pollution, ensuring safe access, and integrating green spaces [2]. The metaverse helps educational institutions meet sustainability goals. In addition, the metaverse provides educators with a digital platform to improve education quality while promoting sustainable development [3, 4]. This immersive virtual space offers practical education solutions and helps students develop key skills, including creativity, critical thinking, teamwork, and communication [5]. Research has shown that these environments enhance engagement in collaborative learning [6, 7]. Moreover, metaverse platforms provide effective settings for both theoretical and practical education [8]. Many educators still use conventional assessment methods that may not effectively match the immersive nature of virtual education [9]. This gap highlights the need for alternative assessment strategies adapted to the dynamics of metaverse-based learning environments.

Parallel to the rise of the metaverse, avatars that serve as virtual representations of individuals have become increasingly significant in educational contexts. The use of avatars in education originated from initial studies on virtual environments and digital self-representation, in which they were described as digital representations of individuals in virtual spaces [10]. This digital representation enables learners to engage in educational tasks beyond the constraints of the physical world [11]. Avatars help students connect more deeply with the subject in virtual environments, encouraging interaction and teamwork [12].

Realistic avatars have the potential to significantly enhance learners’ sense of presence and engagement in virtual environments, particularly in contexts where digital authenticity is highly valued. By closely mimicking human appearances and behaviors, avatars can create immersive and relatable experiences that foster deeper interaction and emotional connection. However, implementing this approach is not without its challenges. When avatars approach a level of realism that is too close to human likeness, they risk triggering a sense of discomfort or unease among users. This phenomenon, often referred to as the uncanny valley effect, occurs when subtle deviations from natural human behaviors or appearances create a sense of eeriness and alienation rather than connection. Consequently, the intended sense of engagement may be undermined, leading to unintended psychological distancing between the user and the virtual environment [13].

This review explores the role of avatars in the educational metaverse (AEM), focusing on their impact on learning, personalized education, and sustainability. This study addresses the following key questions:

- How do avatars improve engagement and interaction in metaverse-based education?
- Key advantages and challenges associated with implementing avatars in personalized and immersive learning.
- Impact of new technologies like generative artificial intelligence (AI) and customization options on learning outcomes and ethical concerns.

This study uses a combined approach, integrating a bibliometric analysis [14] and systematic literature review [15]. Bibliometric evaluation provides a quantitative summary of metadata from a wide range of research articles, covering aspects such as publication year, citations, and authorship. In contrast, a systematic review examines the content to explore detailed insights from selected studies. Together, these methods provide a clear understanding of the theoretical framework and evolving patterns in this field.

The remainder of this paper is organized into five sections. The methodology section outlines the systematic and thematic methods applied to collect and examine relevant literature. The background section provides a historical overview of avatars in education, leading to their current applications within the metaverse. The key themes section discusses technological advancements in avatar design, their impact on education, and the associated challenges and ethical considerations. The discussion section highlights opportunities for innovation, research gaps, and potential contributions to sustainable learning practices. The future research section explores unexplored areas in AI-driven educational avatars, addressing key challenges and opportunities for further innovation. In conclusion, this paper highlights the main findings and provides recommendations for practical applications.

## Methodology

**Fig. 1:**
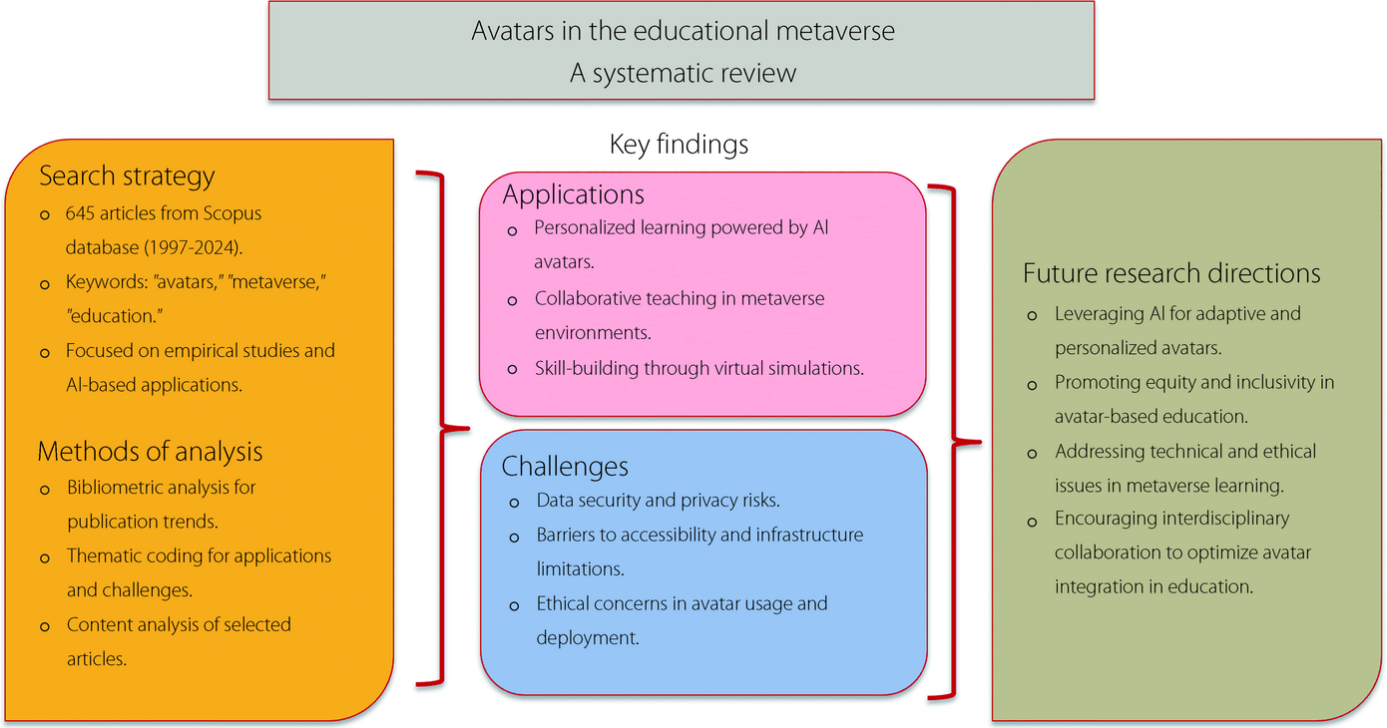
Graphical overview of educational metaverse

Figure 1 provides a systematic review methodology to explore the role of AEM. The data was obtained from the Scopus database and filtered through keyword searches. A mixed-methods approach was utilized, combining bibliometric analysis to identify trends and thematic coding to categorize key findings. A subset of high-quality empirical studies was selected for in-depth content analysis, focusing on applications of avatars in personalized learning, collaborative teaching, and virtual simulations. This study examines challenges in data privacy, accessibility, and ethics, and explores future directions with AI-driven adaptive avatars.

### Data collection

The Scopus database was used to obtain the initial collection of papers for this study. Scopus is a widely recognized resource for performing systematic literature reviews. Using this approach [16], Scopus in November 2024 was searched for English publications containing the terms *avatars*, *metaverse*, and *education* in the title, abstract, or keywords. A total of 1,431 articles were identified through this process.

To refine the dataset, the articles were manually screened to ensure their relevance to the focus on AEM. Publications that lacked substantial content and alignment with the research scope were excluded. Specifically, the following inclusion and exclusion criteria were applied, resulting in a final dataset of 623 articles published between 1997 and 2024.

#### Inclusion criteria

The following criteria were used to select relevant articles for this study:

- Articles published in English.
- Articles containing the terms *avatars*, *metaverse*, and *education* in the title, abstract, or keywords.
- Articles with full-text availability, including journal articles and conference proceedings.
- Articles published between 1997 and 2024.

#### Exclusion criteria

The following criteria were applied to exclude articles from the dataset:

- Articles without substantial content related to avatars in education.
- Articles not aligned with the research scope of AEM.
- Articles lacking empirical data or focusing solely on theoretical perspectives.

**Table 1:** Details of articles included in the dataset

Category	Detail
Year range	1997–2024 (November)
Total journal	278
Total article	623
Average age of article	6.57
Average citation per article	12.86
Total reference	8,010

### Bibliometric data analysis

**Fig. 2:**
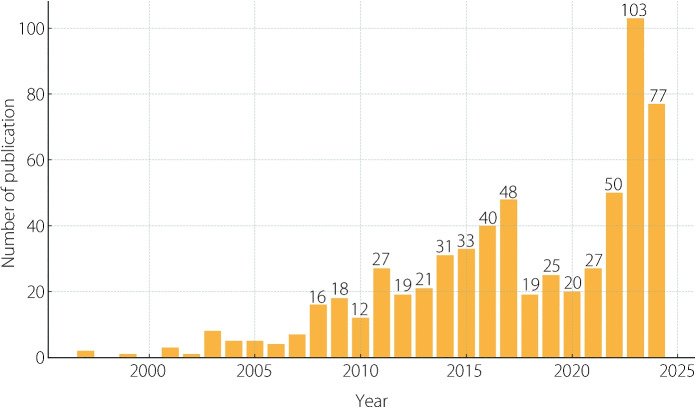
Annual publication trends from 1997 to 2024 (Note: less than 10 are not labeled)

Table 1 provides an overview of the articles in the dataset. These articles are from 278 different journals and are cited in 8,010 publications. The average age of the articles is 6.57 years, showing that most research was published recently, especially in 2015. Figure 2 displays the yearly trend, indicating that research output was relatively low until the early 2010 s. Owing to technological advances and growing global interest in this field, there has been a significant increase in publications from 2017 to 2024.

### Content analysis

Bibliometric analysis is primarily quantitative and excludes a detailed examination of the content within individual research articles. To overcome this limitation, the bibliometric analysis was combined with a systematic review that involved a detailed manual evaluation of a carefully selected subset of studies.

From the initial collection of 623 articles included in the bibliometric analysis, a refined subset was identified for content analysis using the following selection criteria:

- **Publication quality:** Publications appearing in Q1 and Q2 journals according to the Journal Citation Reports, ensuring high-quality sources.
- **Relevance:** Studies explicitly discussing avatars in education and their effects on teaching and learning outcomes.
- **Empirical focus:** Inclusion of empirical studies, which prioritize data-driven insights over theoretical perspectives by addressing specific research questions through measurable evidence.

This focus on empirical studies enables a comprehensive understanding of the AEM research progression and provides actionable insights for future development. A total of 45 articles were selected for detailed content analysis.

**Fig. 3:**
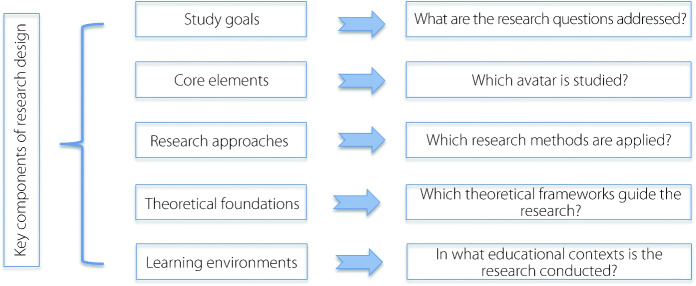
Coding framework for content analysis

Figure 3 presents the coding framework applied to the selected articles. This framework emphasizes the essential aspects of research design, including study goals, core elements, research approaches, theoretical foundations, and learning environments. These components provide a systematic approach for evaluating AEM studies, enabling a clearer understanding of the current landscape, and highlighting opportunities for future investigations.

## Background

The educational metaverse has emerged as a significant innovation in education [17]. As a transformative technology, the metaverse extends beyond education to other sectors, such as entertainment, work, and social life [18]. According to ref. [19], the metaverse improves realism and engagement in virtual learning, enabling students to interact with educators and content beyond the capabilities of traditional digital platforms. The shift toward remote learning has accelerated, positioning the educational metaverse as an essential framework for future-ready education [20]. However, its success depends on how educational institutions can integrate these tools in a manner that is accessible, engaging, and scalable in diverse contexts. This platform brings together a diverse array of resources, including multimedia presentations, interactive tools, and supplementary learning materials, such as videos, images, and audio [21].

The idea of using AI-powered avatars in education began with the creation of the chatbot ELIZA [22], developed by Joseph Weizenbaum in 1966. It uses basic rules and keyword-matching methods to simulate human conversations. Although ELIZA’s replies were simple, they successfully made many users believe that they were engaging in meaningful conversations. This success demonstrated the potential of conversational agents to emulate human interactions, albeit with a limited capacity.

In 1995, ALICE, one of the first chatbots, was introduced. Based on AI principles, ALICE uses artificial intelligence markup language to process and respond to user input, which leads to more dynamic and relevant conversations [23]. In contrast to ELIZA, which relies solely on static rules, ALICE includes a large natural language library and customizable knowledge base. This design allows for a more convincing simulation of human responses, and has become an important step in the development of chatbots.

Although these early advancements were fundamental, many chatbots, particularly in the education sector, remained primarily text-based and relied on simple algorithms. They could not process complex natural language and adapted effectively to diverse educational contexts. Despite these limitations, chatbots established the foundation for integrating AI into education. They eventually led to the development of AI-powered avatars that combine advanced natural language processing, machine learning, and multimodal interaction capabilities [24].

In 2012, AlexNet achieved a breakthrough in deep learning using neural networks, with impressive results in image classification tasks [25]. This success demonstrated the potential of large-scale models, which are now widely used for advanced conversational AI, such as ChatGPT [26]. At the same time, animated pedagogical agents, such as *Steve*, began appearing in virtual reality (VR) training environments. Steve was designed to guide learners through complex tasks, including naval operations, by demonstrating steps, providing feedback, and helping with teamwork activities [27]. Another major step was AutoTutor, a system that used conversational dialogue to help students learn. It could interpret text inputs, detect errors in understanding, and adapt its responses to provide helpful feedback, making learning more interactive and personalized [28].

Empirical research has shown that pedagogical agents can improve learning; however, meta-analyses and reviews have found that their impact on knowledge and emotional outcomes is moderate [29, 30]. Despite these limitations, this evolution highlights its role in the future of educational technologies.

## Key themes

### Technological advances in avatar design

Technological advances in natural language processing, automatic speech recognition, and text-to-speech systems have significantly improved the capabilities of virtual learning agents in 2016 [31]. These agents have become much more advanced, able to understand what students say and respond naturally, similar to humans. Marni, a virtual science tutor, used spoken dialogues to help students learn [32]. It processed students’ answers, provided feedback, and used animations and simulations to make science topics easier to understand. These agents not only guided students but also supported their learning by acting as helpful partners in interactive educational experiences [27].

Recent breakthroughs in AI have enabled the development of advanced educational avatars powered by large language models (LLMs) and generative AI models. These avatars can represent figures similar to humans, interact within immersive virtual environments, and follow complex educational prompts. While many of these capabilities existed in earlier systems, significant improvements in AI reliability, speed, and integration have advanced these avatars to a new level of development. Consequently, avatars now combine the strengths of earlier chatbots and pedagogical agents with modern generative AI technologies. The GPTAvatar [33], a cutting-edge AI-driven avatar, captures user speech input through a microphone, converts it to text using speech recognition, and processes responses using an LLM. Responses were transformed into realistic synthetic speech, synchronized with lip movements on a 3D avatar for a highly authentic user experience. In addition, the avatar was embedded within a customizable 3D virtual environment, with adjustable personality traits and response behaviors configured via a settings file. Built using the Unity game engine, GPTAvatar is open source, enabling developers to create custom educational avatars designed for specific use cases.

Modern LLMs, including GPT-4, have demonstrated the potential to enhance learning and problem-solving capabilities. Studies evaluating ChatGPT’s performance, such as its ability to complete the United States Medical Licensing Examination, highlight its capacity to process complex textual and contextual data effectively [34, 35]. In addition, empirical research, such as meta-analysis, revealed that chatbots can significantly affect learning outcomes, with moderated results based on task-specific implementations and experimental conditions [36].

### Avatars for teaching and learning

Avatars have three principal roles in educational contexts: supporting learning, facilitating tasks, and mentoring students. As learning tools, they deliver and assess knowledge using personalized and adaptive methods. In assisting learners, avatars simplify processes, provide guidance, and help address immediate challenges during educational activities. As mentors, these AI systems support development by building skills, encouraging self-regulation, and adapting to individual needs [37].

#### Personalized learning

AI-enabled avatars have emerged as powerful tools for delivering highly personalized and adaptive learning experiences. A significant application of these avatars is in teaching foreign languages, where technologies such as automatic speech recognition and text-to-speech systems enable interactive, real-time conversations in multiple languages. These capabilities make them valuable for language learners who require practice and immediate feedback [38]. Beyond language instruction, avatars are increasingly used in science and engineering education, helping to break down complex concepts and adjust content delivery to adapt to individual learning needs. In addition, they offer specialized assistance to learners encountering particular educational difficulties by modifying their feedback, promoting participation, and implementing reinforcement techniques to maintain motivation [31].

Personalization features inherent in AI-driven avatars function using two fundamental mechanisms. Students can customize lessons by choosing their preferred teaching methods, content difficulty, and focus areas, giving them control over their learning. However, these avatars can dynamically adjust instructional content by assessing student progress and performance in real time. AI technologies, such as generative models and multimodal learning analytics, further increase this adaptability by making sense of data derived from user interactions, behavioral trends, and emotional feedback. This leads to the optimization of lesson delivery, thus making it more engaging and effective [39]. Furthermore, design elements, such as facial expressions and culturally appropriate appearances, are also important for maintaining user engagement and comfort during the learning experience [40].

These avatars can create engaging and participative learning environments. For example, they can simulate real-world activities in virtual environments, offering hands-on experiences in the area of science, technology, engineering, and mathematics (STEM) education and professional training [32, 41]. By combining adaptive features with engaging virtual worlds, AI-based avatars provide a highly personalized and immersive learning experience, making them an essential tool for modern education [39].

#### Contextualized teaching

Intelligent avatars are revolutionizing contextualized teaching by offering interactive environments in which learners can connect their theoretical knowledge to real-world scenarios. These avatars enable students to engage in complex, resource-intensive activities, including role-playing and case-based simulations, which are traditionally expensive and difficult to implement [42]. Compared with traditional methods, avatars are cost-effective and flexible. They provide dynamic responsiveness by adapting to user input, creating highly personalized learning experiences [43].

In medical education, avatars simulate patient interactions, allowing learners to practice diagnosing conditions, improve communications, and receive real-time feedback. This aligns with case-based learning principles in which students analyze and respond to specific situations to build critical thinking skills [44]. In teacher training, avatars replicate classrooms in which educators can improve strategies, manage dynamics, and practice handling diverse student behaviors. These virtual instructors use gesture-based teaching, mimicking traditional classrooms with nonverbal cues to improve cognitive processing and knowledge retention. By creating realistic and engaging scenarios, avatars help educators develop problem-solving and decision-making skills while bridging the gap between virtual and in-person instruction. This approach has been shown to significantly improve engagement and learning outcomes [45–47]. Through avatar-based simulations, educators participate in realistic classroom scenarios [48]. It also allows teachers to practice managing diverse student behaviors, experiment with instructional strategies, and build confidence in a controlled and consistent environment.

AI-based avatars also offer significant scalability in educational settings. By integrating speech recognition and natural language processing, avatars can adapt lessons to the learner’s progress in real time. This personalized approach ensures that each student receives the appropriate level of challenge and feedback [43]. Research shows that avatars are not only effective in role-playing scenarios but also enhance communication and decision-making skills, enhancing the learner’s understanding of the subject matter [49].

#### Virtual learning

Virtual learning utilizes technologies including virtual, augmented, and mixed reality to create deeply engaging and realistic environments for learners. These environments not only provide opportunities for active participation, but also present challenges, such as increased cognitive load and difficulty in navigating complex virtual spaces [50]. Avatars help address these challenges by guiding learners, reducing their mental effort, and providing real-time feedback to keep users focused and engaged. By acting as adaptive navigational aids, they can enhance the overall learning experience in these dynamic settings.

Traditional knowledge acquisition strategies are often inadequate in virtual learning because learners may struggle to use familiar techniques, such as summarizing, self-testing, and visualizing, in highly interactive environments [51]. To address these challenges, avatars can promote generative learning strategies, such as creating concept maps, explaining ideas aloud, and teaching concepts, which have been shown to improve memory and understanding [52]. For instance, they can offer structured prompts and guide learners’ thinking processes, making these strategies more accessible and actionable in complex virtual environments [53]

In addition, avatars in virtual learning environments enable learners to engage in tasks that require collaboration, exploration, and problem solving [54]. Metaverse platforms improve these experiences by combining interactive elements with engaging features, such as challenges and rewards, to increase participation [5].

### Challenges and ethical considerations

#### Hallucinations and trust in AI

LLMs are powerful tools that generate coherent and contextually relevant responses. However, these can also produce outputs that contain misleading or factually incorrect information—a phenomenon commonly referred to as hallucination [55]. These hallucinations pose significant challenges, particularly in applications in which accuracy and reliability are paramount, such as education, healthcare, and decision-making systems. The issue arises when LLMs generate plausible sounding but incorrect information, often due to gaps in the training data, ambiguous input contexts, or limitations in the model’s understanding of complex queries.

A key factor contributing to hallucinations is the limited context window of LLMs, which can cause them to forget earlier parts of a conversation or fail to fully grasp the nuances of a query. This limitation can lead to inconsistent or inaccurate responses, even when the model is trained on high-quality data. In addition, the training data itself may contain biases, stereotypes, or inappropriate values [56], which can further exacerbate the problem. For instance, if an LLM has been trained on a text corpus that includes harmful or biased material, it may inadvertently generate outputs that reflect inappropriate values, even if the information is factually incorrect.

The challenge of hallucinations is further compounded by the manner in which information is presented to users. AI systems that employ photorealistic avatars, realistic speech patterns, and expressive body language can create an illusion of credibility, even when the information being conveyed is inaccurate. Research has shown that users are more likely to trust information presented by AI avatars that exhibit human-like characteristics, such as unique personalities and emotional expressions. This phenomenon can lead to a situation where users are convinced of the accuracy of false or misleading information simply because it is delivered convincingly [57].

#### Data security and privacy

A significant challenge in the deployment of advanced AI technologies, such as LLMs and generative AI systems, lies in ensuring robust data security and privacy. These systems often rely on cloud-based infrastructures operated by companies across multiple jurisdictions, leading to inconsistencies in data protection regulations and compliance standards. This fragmented regulatory landscape creates vulnerabilities that can be exploited, particularly when sensitive information is involved. For instance, technologies such as automatic speech recognition and deepfake algorithms, powered by generative adversarial networks, can misuse personal data such as voice recordings or images to create convincing but fraudulent content. This not only raises concerns about identity theft but also contributes to the spread of misinformation, undermining trust in digital systems [58].

In educational settings, where AI-based avatars and LLMs are increasingly used to provide personalized learning experiences, the risks to data security and privacy are particularly pronounced. Students interacting with these systems may unknowingly disclose personal or confidential information, such as academic performance, career aspirations, or even emotional and psychological challenges. If mishandled, these data could be exploited for commercial purposes or leaked to unauthorized parties, leading to significant ethical and legal ramifications [59]. Moreover, AI avatars that offer counseling or adaptive instruction often collect highly sensitive information about learners, including their strengths, weaknesses, and personal struggles. Such data must be handled with the utmost care to maintain confidentiality and trust, particularly when dealing with young or vulnerable users.

#### Accessibility and infrastructure

The accessibility and technical requirements of metaverse platforms present significant challenges in educational environments, particularly in ensuring equitable access and effective implementation. For educational experiences in the metaverse to be successful, they rely heavily on an advanced infrastructure capable of supporting high-speed networking, decentralized data exchange, and reliable hardware. These prerequisites are essential to deliver a seamless, immersive, and interactive learning experience. However, the high costs and technical complexity associated with such infrastructures often create barriers that make it difficult for institutions, particularly those in resource-constrained areas, to adopt these technologies [20].

The need for advanced networking capabilities, such as low-latency connections and high bandwidth, are critical for ensuring smooth interactions within the metaverse. Without these, users may experience delays, disruptions, or reduced quality of engagement, which can significantly hinder the learning process. In addition. decentralized data exchange mechanisms, which are often integral to metaverse platforms, require sophisticated technical expertise to implement and maintain. This further increases the complexity and cost of deployment, making it a challenge for schools and universities with limited information technology resources to adopt such systems [17].

## Discussion

Examining the role, impact, and future directions of avatars within the educational metaverse suggests many possibilities for continued research and development. With their advancement, digital avatars can transform education by providing sustainable and accessible learning experiences aligned with global educational goals. Pedagogical agents are increasingly recognized as important elements in modern learning environments. These agents act as virtual tutors or partners, providing individualized support to learners by adapting to their needs. They can provide immediate feedback, guide students through different activities, and even recognize emotions such as frustration, and offer encouragement. Advances in technology have made these agents more engaging and effective. However, the processes of developing and customizing them must be made easier for teachers. Facilitating their accessibility and adaptability is essential for increasing their application in educational settings and training programs [31].

The integration of state-of-the-art technologies, such as VR and augmented reality (AR), within educational metaverse models is a significant advancement in technological innovation. These technologies enhance simulation-based, hands-on learning experiences, particularly in fields that require practical engagement, such as STEM, medicine, and environmental science. Studies have demonstrated the effectiveness of VR and AR in improving cognitive engagement and content retention, making them particularly valuable for complex and inaccessible learning scenarios [17, 60]. Embedding these technologies into avatar-based environments enables students to engage in real-time problem solving and critical thinking.

Recent advancements, including GPT-4o and collaborative generative agents, have redefined the potential of AI in dynamic, multi-agent environments [61]. GPT-4o introduces enhanced capabilities such as processing multimodal inputs, including images and videos, as well as advanced emotion detection and computer vision skills. These features open new possibilities for the creation of highly interactive and responsive systems. Similarly, research on generative agents has shown how AI can simulate human-like behaviors, including reasoning, teamwork, and real-time adaptation. For example, in simulated environments such as job fairs, agents can interview, recruit, and coordinate tasks via perception, memory, reasoning, and execution modules [62]. The combined power of GPT-4o and socio-emotional intelligence could become powerful systems for education and workplace collaboration. However, some challenges remain in the areas of goal misalignment and contextual misunderstandings, which still require development. With the future development of AI, integration with generative systems will allow for more efficient collaboration and decision-making.

## Future research

Several areas exist where AI-based educational avatars have not been fully explored, offering promising avenues for future research. One critical area is the integration of digital twins and real-world data into educational applications. Although avatars can process data from these sources, there is a need to investigate how such data can be meaningfully utilized to enhance learning experiences. For instance, real-time data from the Internet of Things devices or simulations can be leveraged to create adaptive, context-aware learning environments that respond dynamically to student needs. This could involve exploring how avatars can act as intermediaries between students and complex datasets, simplifying information and guiding learners through real-world problem-solving scenarios.

Another important gap lies in understanding how students trust and emotionally connect with avatars, particularly in long-term or emotionally sensitive educational contexts. Research should explore the psychological and social dynamics of avatar-student interactions, especially in scenarios where avatars are used for counseling, mental health support, and personalized mentoring. For example, how do students perceive avatars that exhibit human-like emotions, and what factors influence their willingness to engage with these virtual agents over extended periods? Investigating the role of cultural and individual differences in avatar acceptance could also provide valuable insights into the design of more inclusive and effective educational tools.

Ethical considerations remain a significant challenge in the deployment of AI-driven avatars. Key questions include how to use avatars responsibly with children and vulnerable populations, ensuring that these technologies do not inadvertently cause harm or perpetuate biases. In addition, the risk of misinformation and hallucinations generated by LLMs must be addressed. Future research should focus on developing robust mechanisms to detect and mitigate inaccuracies in avatar-generated content, as well as establishing clear guidelines for the ethical collection and use of sensitive student data. This includes exploring ways to balance the benefits of personalized learning with the need to protect student privacy and autonomy.

Furthermore, the scalability and accessibility of avatar-based education systems require further investigation. Although these technologies hold great potential, their implementation is often hindered by high costs and technical requirements. Future studies should explore methods to make avatar-driven learning platforms more accessible to resource-constrained regions to ensure that the benefits of these technologies are equitably distributed. This could involve developing lightweight, low-cost solutions or investigating how avatars can be integrated into existing educational infrastructures.

## Conclusions

This study examined how AEM support immersive learning, collaboration, and skill development. Although the advantages are clear, challenges with regard to data privacy, ethical issues, and technical limitations must be addressed for the effective use of avatars in education. Future research should focus on increasing accessibility, building trust, and creating ethical guidelines for safe and responsible use. With further development, avatars can make education more flexible, accessible, and enjoyable for both learners and teachers.

## Data Availability

Not applicable.

## Abbreviations

3D: Three-dimensional
AI: Artificial intelligence
AEM: Avatars in the educational metaverse
VR: Virtual reality
LLM: Large language model
STEM: Science, technology, engineering, and mathematics
AR: Augmented reality
